# Exploratory Analysis of Urban Climate Using a Gap-Filled Landsat 8 Land Surface Temperature Data Set

**DOI:** 10.3390/s20185336

**Published:** 2020-09-17

**Authors:** Sorin Cheval, Alexandru Dumitrescu, Vlad-Alexandru Amihaesei

**Affiliations:** 1 Meteo Romania (National Meteorological Administration), 013686 Bucharest, Romania; dumitrescu@meteoromania.ro (A.D.); vlad.amihaesei@meteoromania.ro (V.-A.A.); 2 Henri Coandă Air Force Academy, 500187 Brașov, Romania; 3 Research Institute of the University of Bucharest, 050663 Bucharest, Romania; 4 Romanian Association of Applied Meteorology and Education, 010041 Bucharest, Romania; 5 GeoScience Doctoral School Department, “Alexandru Ioan Cuza” University, 700506 Iasi, Romania

**Keywords:** land surface temperature, urban climate, Landsat 8, filling missing data, dineof, Bucharest

## Abstract

The Landsat 8 satellites have retrieved land surface temperature (LST) resampled at a 30-m spatial resolution since 2013, but the urban climate studies frequently use a limited number of images due to the problems related to missing data over the city of interest. This paper endorses a procedure for building a long-term gap-free LST data set in an urban area using the high-resolution Landsat 8 imagery. The study is applied on 94 images available through 2013–2018 over Bucharest (Romania). The raw images containing between 1.1% and 58.4% missing LST data were filled in using the Data INterpolating Empirical Orthogonal Functions (DINEOF) algorithm implemented in the *sinkr* R packages. The resulting high-spatial-resolution gap-filled land surface temperature data set was used to explore the LST climatology over Bucharest (Romania) an urban area, at a monthly, seasonal, and annual scale. The performance of the gap-filling method was checked using a cross-validation procedure, and the results pledge for the development of an LST-based urban climatology.

## 1. Introduction

The increasing population and the permanent quest for comfort and safe shelter have triggered intense urbanization processes taking place all over the world, mainly in the 20th and 21st centuries. The built-up areas substantially modify the environment, and the features of the local atmospheric envelope are changed to the point that a new type of climate is formed. The urban climate is the resultant of a different composition of the radiation budget, higher temperature, and lower humidity values than the surrounding rural areas. The high heterogeneity of the urban environment is replicated in a corresponding diversity of the local climate conditions within a city. For example, green areas are cooler and more humid than impervious patches, urban canyons and squares disturb the wind flow, and building heights and density are so influential for the urban climate that they form the base of the definition of local climate zones (LCZs) [1,2,3]. As a consequence, urban climate modelling and weather forecasting require adequate data relevant for different urban microclimates.

Due to some insurmountable challenges related to the deployment of sensors, such as implementation and maintenance costs, it is very difficult to capture the climate of all distinct spatial tracks in a city using ground measurements. In recent years, urban meteorological networks (UMNs) have been implemented in several cities [4,5,6,7], and citizen observatories are increasingly used in urban climate research [8,9], but a full coverage of the climatic instances of a city is still problematic. Alternatively, the use of satellite remote sensing for retrieving the urban land surface temperature (LST) has been constantly intensified in the last decades, urged by easier access to low-cost ready-to-use products, larger spatial coverage, finer temporal resolutions, and an extended time span of availability. At present, the World Meteorological Organization (WMO) acknowledges 105 satellite instruments that have provided LST along time, categorized from primary to marginal relevance [10]. Several sensors that have provided LST products are particularly useful for the urban climate due to their suitable resolutions and time span (Table 1).

From a climatic perspective, the main differences between the satellite remote sensing products refer to the spatial-temporal resolutions and time span of available data. For urban climate studies, the ideal product will have ≤ a 1-km spatial resolution [11], daily frequency, and temporal continuity over the area of interest. In this respect, one can remark that the Landsat missions jointly operated by NASA and the U.S. Geological Survey have provided a series of Earth observation satellites since 1972. The spatial resolution of the Landsat series reached 30 m (resampled) and contributed significantly to the development of urban climate research [12,13,14]. The availability of such data has stimulated detailed investigations of the surface urban heat island (SUHI) and its correlation with the land use/land cover [15,16]. Nevertheless, the low frequency of full-coverage images, with all or the majority of pixels valid over certain areas, and the coarse temporal resolution (i.e., one image at 8 or 16 days) bias the potential benefits to use the Landsat series for urban climate research. Different solutions have been proposed to address such limitations or to improve the quality of the outputs. Cristóbal et al. (2009) [17] presented an enhanced methodology to retrieve LST from Landsat 4 TM, Landsat 5 TM, and Landsat 7 ETM+ using different water vapor ranges.

Sparse or even singular Landsat images were often used in urban climate studies [14,15], while temporally and spatially continuous data sets have been rarely employed simply because they are not available. For example, Lemus-Canovas et al. (2020) [18] estimated Barcelona’s metropolitan daytime hot and cold extremes using the LST retrieved from 24 Landsat 8 imagery, Tsou et al. (2017) [15] assessed the SUHI of Shenzen and Hong Kong based on 4 Landsat 8 images, and Kaplan et al. (2018) [14] explored the case study of the Skopje SUHI using only 2 July images. The value of such investigations on the urban climate is doubtless, but one should also acknowledge the shortcomings of the results due to the limited number of samples.

Comparatively, this study addresses the need for temporal and spatial continuity of remote sensing data, and it explores the climatology of the LST in a large urban area (Bucharest, Romania) based on 94 high-spatial-resolution images retrieved from Landsat 8. We used the Data INterpolating Empirical Orthogonal Functions (DINEOF) algorithm [19] in order to solve the inherent problem of missing data within satellite images, so that the exploratory study is ultimately based on a gap-free data set. Zhou et al. (2017) [20] showed that the DINEOF reconstruction method can capture the impact of land cover types on LST, pledging for a reasonable spatial pattern, which is particularly important in urban climatology. The temporal extent and the data objectively available for this study support a methodological approach, which can certainly be extended when more data are collected in the future over the same area. In the end, the seasonal and annual variations of the LST were explored.

High-spatial-resolution data sets with full coverage over an urban area have multiple applications beyond scientific research. For example, the temperature distribution over smaller administrative units within a city can provide useful information for urbanism, health risk assessment, or building industry. In this respect, we illustrate the distribution of the LST values over census units of Bucharest.

The manuscript is structured in four sections, as follows: After the introduction (1), we briefly present relevant geographical and climatic facts about the study area, and we detail the data and methods (2), which fundament the results (3), and we supply a set of concluding remarks (4).

## 2. Materials and Methods

### 2.1. Study Area

**Figure 1:**
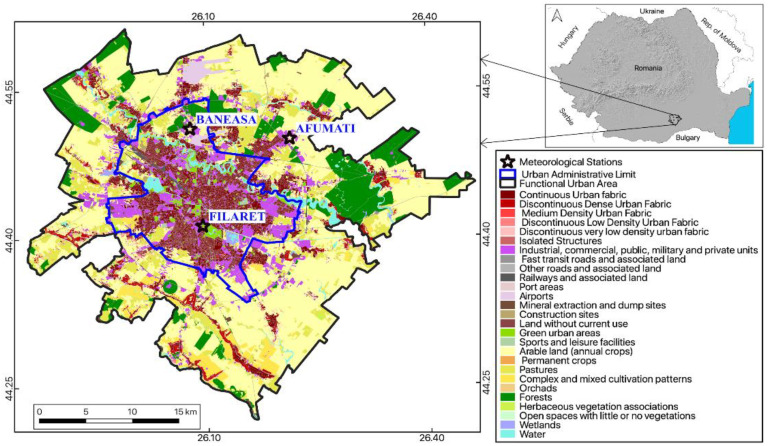
Urban administrative limit and functional urban areas (FAU) of Bucharest. (Source: Urban Atlas LCLU 2012, available online at <https://land.copernicus.eu/local/urban-atlas/urban-atlas-2012>).

**Figure 2:**
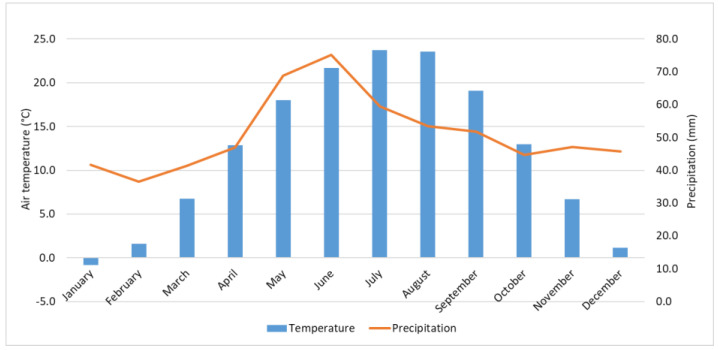
Average monthly air temperature and precipitation amounts at Bucharest (1961–2018).

The case study focuses on Bucharest, the capital and the largest city of Romania, with an estimated population of about two million inhabitants [21], lying over approximately 240 km^2^. The climate of Bucharest is monitored by three WMO stations, namely one ‘urban’ weather station, i.e., București-Filaret, placed within the city limits; and two ‘peri-urban’ weather stations, i.e., București-Băneasa, at 10 km N of the downtown, and București-Afumați, at approximately 11.5 km NE of the downtown (Figure 1). According to the Köppen–Geiger climate classification [22], Bucharest has a hot-summer humid continental climate (Dfa) with the coldest month, January, averaging below 0 °C, while in July and August, the average temperature is above 22 °C, and above 10 °C from April to October. The wettest period is April–July (Figure 2).

### 2.2. Data and Methods

The Landsat 8 mission provides timely high-quality visible and infrared images, sufficiently consistent with other satellite data in terms of acquisition geometry, calibration, coverage, and spectral characteristics [23]. We used the LST derived from the bands 10 (10.60–11.19 µm) and 11 (11.50–12.51 µm), resampled at 30 m, from Landsat 8 TIRS (Thermal Infrared Sensors) instruments. The Landsat 8 TIRS products use split window algorithms and techniques for correcting atmospheric disturbances, such as absorption and emission, or surface emissivity inferred from MODIS land-cover calculations. Emissivity is a critical variable for the LST estimation. This study used Landsat 8 LST with NDVI-based emissivity, estimated from the Landsat visible and near-infrared bands and typical emissivity values, retrieved from http://rslab.gr/downloads.html, with full technical details available online [13].

The scenes were retrieved between 08:58 and 09:04 UTC, and the area of interest covers the administrative perimeter of Bucharest and its surroundings within a rectangle ranging between 25.90° and 26.32° East, and 44.32° and 44.59° North. This study was based on 94 Landsat 8 imagery ranging between 2013 and 2018, with an average percentage of data coverage of 91.4% (Table 2). Table 3 shows the degree of completeness of the 94 images when downloaded. In order to secure the quality of the results and to minimize the sampling and processing errors, the data beyond the 1st and 99th percentiles were filtered out.

Missing data is a major barrier for satellite meteorology and especially for climate applications, which require long-term and good spatial coverage of information, and constant efforts address this issue. Henn et al. (2013) [24] compared the performance of five techniques used to fill in missing temperature data, namely (a) spatiotemporal correlations based on empirical orthogonal functions (EOFs), (b) time series diurnal interpolation, and (c, d, e) three variations of lapse rate-based filling. They found that the spatiotemporal correlations using EOF reconstruction were most accurate for a large number of stations and missing data. Long et al. (2020) [25] combined MODIS and SEVIRI LST in order to generate a complete daytime data set, and Zhao et al. (2020) [26] also used MODIS to obtain all-weather conditions data. However, high-spatial-resolution data sets allow in-depth analysis of the LST at an urban and intra-urban scale. Considering the results provided by Henn et al. (2013), this study addressed such limitations by reconstructing complete LST data sets for the selected Landsat images through 2013–2018 using the DINEOF algorithm [27,28,29]. The selection of this method was justified by its relatively simple application in situations when relevant co-variables are not available.

Essentially, the missing LST values of each Landsat 8 image were reconstructed using the function DINEOF implemented in *sinkr* R package [30]. This method does not require *a priori* knowledge about the statistics of the full data set [31], which is an advantage for analyzing extensive data sets.

The DINEOF method consists of an iterative method to calculate the field values at missing positions. It implies the application of the following routine [19,29,32]:

1. The spatial and temporal mean of the observation data are removed from the raw LST dataset.
2. The ‘no observation’ pixels are replaced with zero values.
3. The resulting dataset is used to compute the first EOF, and the values obtained during the EOF decomposition are used to replace the missing data.
4. Sequential EOFs are calculated iteratively until a user-defined convergence criterion is reached.
5. The procedure is repeated by computing the two EOFs, three EOFs, etc.
6. The total number of EOFs is determined by the results of the cross-validation procedure, commonly checked with 1% of the valid data selected at the beginning of the procedure.

**Figure 3:**
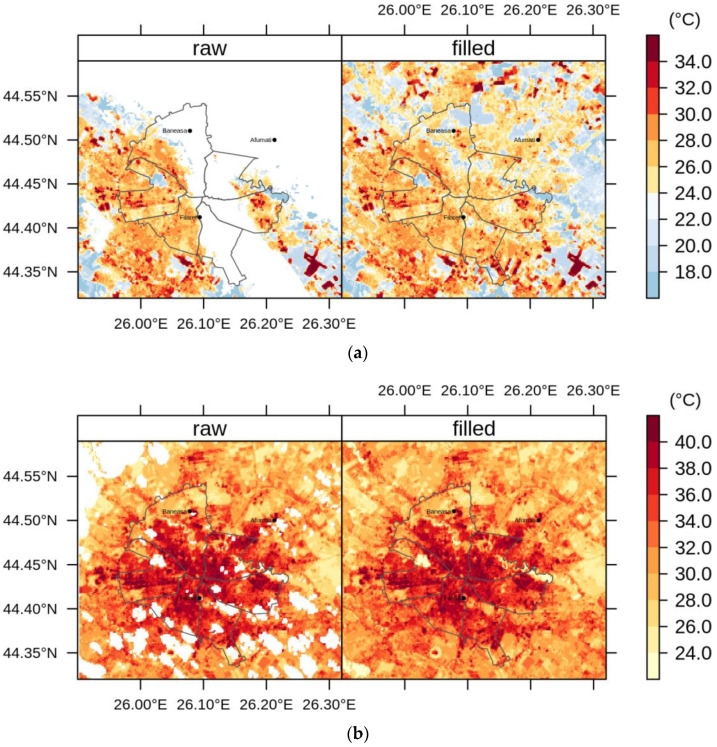
Land surface temperature (LST) (°C) over Bucharest (Romania) as retrieved from raw and gap-filled Landsat 8 images for 23 April 2013 (**a**) and 25 June 2016 (**b**). White spots correspond to missing data.

The use of only one selected EOF configuration may be considered a limitation of the DINEOF algorithm [33,34], but the accuracy of the results reported here pledge for applicability in urban climate research. Overall, the cross-validation pixels were utilized to calculate the accuracy by comparing the pixels of the raw data sets with the pixels of the filled data sets. Figure 3 exemplifies the visual performance of the DINEOF method for filling in the missing LST values of two Landsat 8 images over the urban areas of Bucharest (Romania).

**Figure 4:**
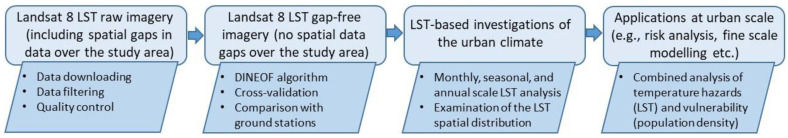
Workflow from raw Landsat 8 LST data to applications based on gap-free LST in an urban area.

The complete working flow of this study, from retrieving the Landsat 8 LST images to potential applications of the resulted gap-free data set, is provided in Figure 4. The approach was applied here for the city of Bucharest, but it is clear that a similar flow would be applicable for any urban area, and various applications may be proposed.

## 3. Results and Discussion

### 3.1. Gap Filling the Landsat 8 Land Surface Temperature Data Set: Results and Validation

**Figure 5:**
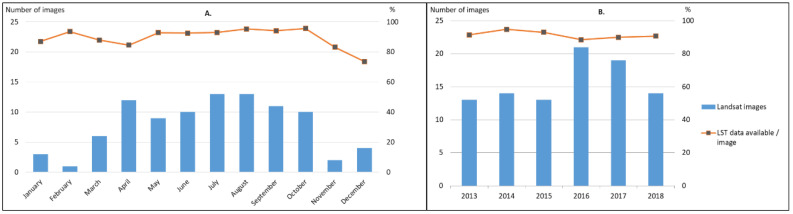
Absolute and relative frequencies of Landsat 8 LST images and data available over the study area through 2013–2018: monthly (**A**) and annual (**B**) statistics.

By applying the DINEOF gap-filling method, we obtained 94 Landsat 8 images with full coverage of the study area. Between 2013 and 2018, the annual number of available images ranges between 13 and 21, while the monthly number of images varies between 2 in February, and 13 in July and August (Figure 5). More frequent images are available during the warm season (i.e., 9 to 13 images from March to October) due to the lower cloudiness.

**Figure 6:**
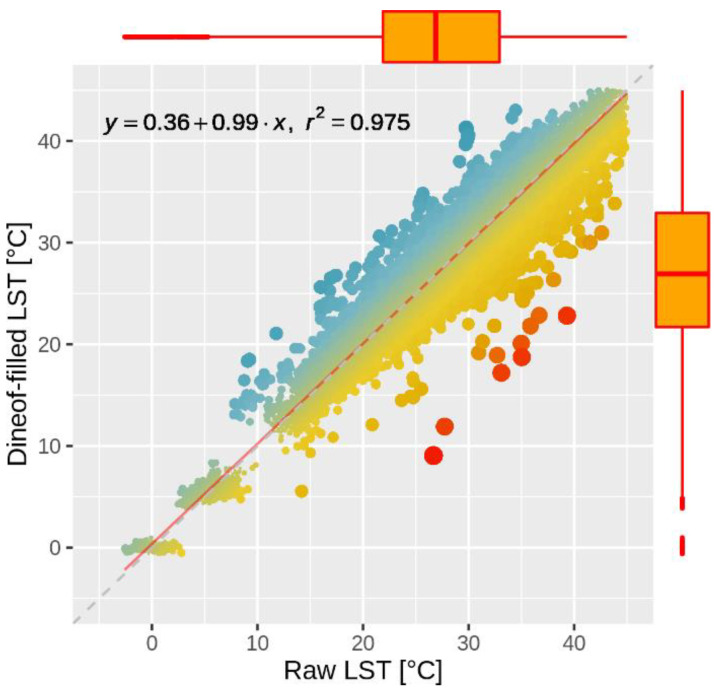
Comparison between DINEOF-filled LST and raw LST data over the validation sample. The boxplots depict the statistical properties of the two data sets, i.e., median, 25th, 75th percentile (the lower and upper hinges), and the 1.5 multiplied with the interquartile range (the upper and the lower whiskers).

In order to evaluate the accuracy of the DINEOF gap-filling method, we artificially simulated gaps in the original LST data set, and the estimated values were compared to the raw LST values. The artificial gaps were created on a subset representing 50% of the selected LST images, and 1000 randomly selected pixels were assigned as not available (NA) for each image. The DINEOF gap-filling method was tested on the entire data set, namely on all the images containing artificially created gaps. Figure 6 shows the relationship between estimated and original LST values. One can notice that the DINEOF algorithm results in a gap-filled LST data set that is very well correlated and statistically consistent with the initial LST data set, i.e., Pearson’s correlation coefficient *r*^2^ = 0.979, and only −0.3 °C difference between the average values (Table 4).

**Figure 7:**
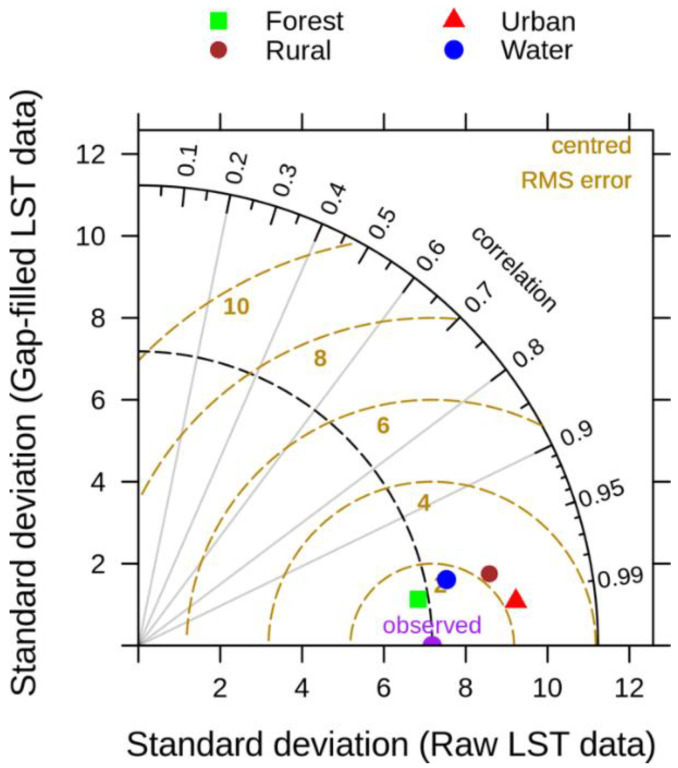
Statistical summary of the matching performance obtained using the DINEOF gap-filling method. The gap-filled data set was compared to observations using a validation subset containing 50% of the selected LST images, and 1000 randomly selected pixels assigned as NA for each image.

The performance of the matching between the gap-filled and raw LST data sets was also summarized in terms of the correlation, root-mean-square (RMS) errors, and amplitude of their variance (standard deviations) using a Taylor diagram [35], computed for four distinct land-cover categories derived from the Urban Atlas LCLU 2012 (https://land.copernicus.eu/local/urban-atlas/urban-atlas-2018), namely urban, rural, forest, and water (Figure 7). One can indicate the following deduction: (a) The performance of the DINEOF method is very good and similar for the land-cover categories (i.e., very high correlation coefficients, and low RMS errors); (b) based on the standard deviation values, the gap-filled data are closer to the raw data for land-cover categories closer to nature (i.e., forest and water) than for the more anthropic ones (i.e., rural and urban); and (c) there is a clear distinction between the more natural categories and more anthropic ones in terms of RMS errors, but the values are low in all the cases (±2.0 °C).

### 3.2. Climatic Analysis of the Land Surface Temperature over Bucharest Using the Gap-Filled Landsat 8 Data Set

Climate research requires long-term data and full spatial coverage, if possible. While a 30-year period is recommended for climate prediction purposes, shorter time intervals may perform as effectively as 30-year averaging periods and provide useful overall climate information [36]. It is undoubtedly an advantage to have early climate information over an area even if it is consequently from shorter periods, than to delay climate research and wait for the completion of a 30-year period of data. In this respect, the spatial completeness and the temporal extent of the reconstructed Landsat 8 LST data series supports a preliminary climatic analysis over Bucharest at monthly, seasonal, and annual scales.

**Figure 8:**
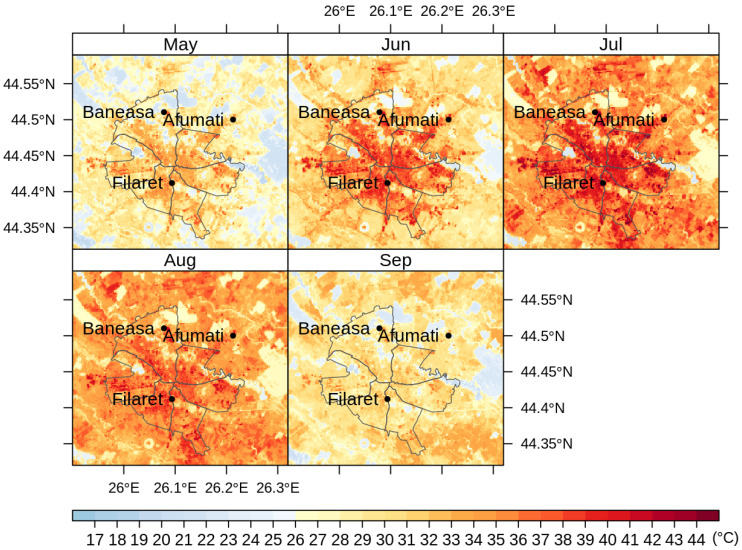
Average monthly LST values (°C) over Bucharest retrieved from Landsat 8 imagery (2013–2018).

**Figure 9:**
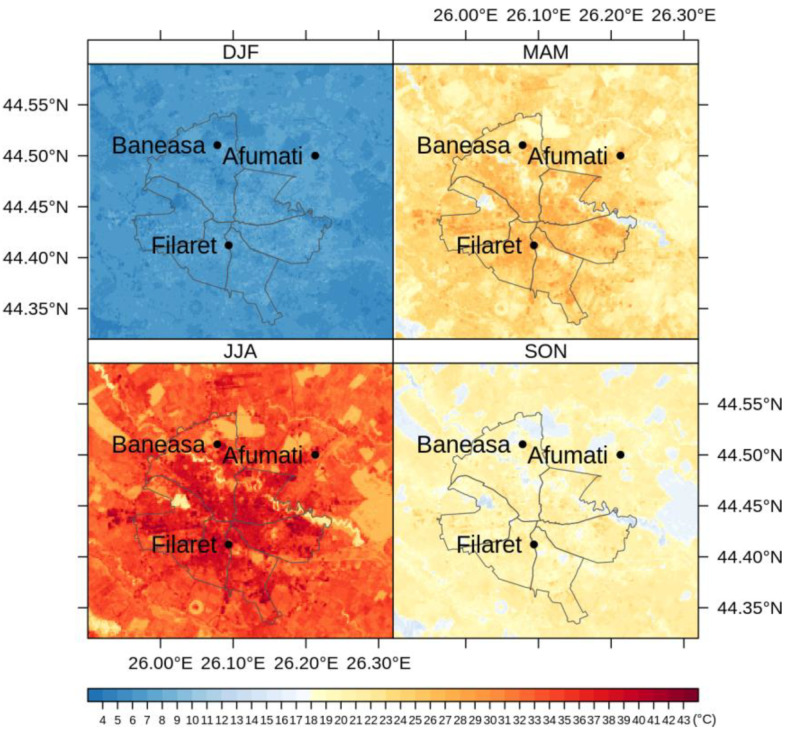
Average seasonal LST values (°C) over Bucharest retrieved from Landsat 8 imagery (2013–2018).

**Figure 10:**
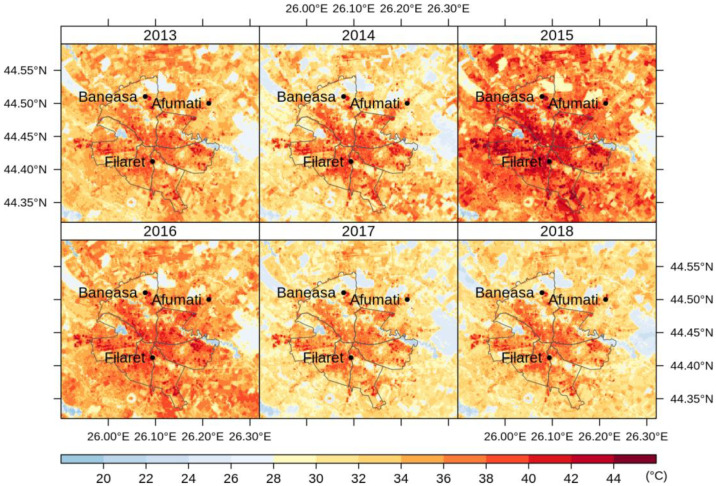
Average summer LST values (°C) over Bucharest retrieved from JJA Landsat 8 imagery (2013–2018).

**Figure 11:**
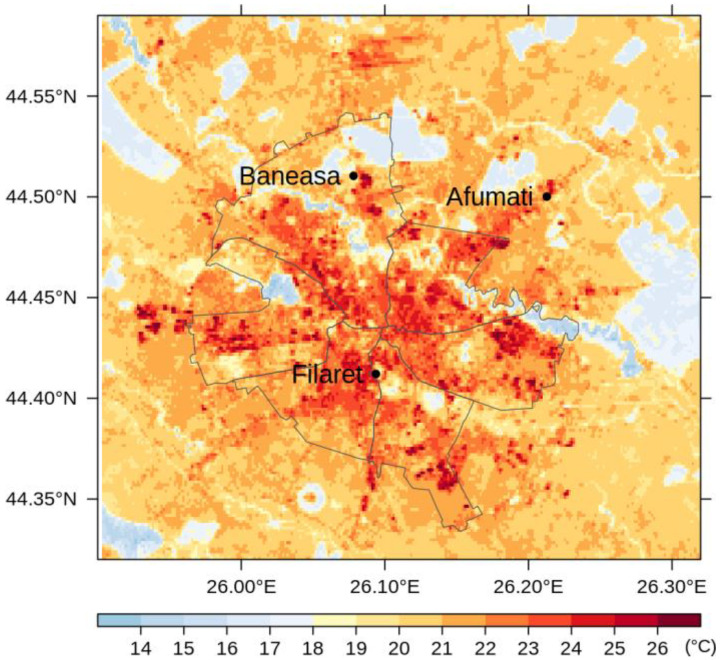
Multi-annual average LST over Bucharest during the warm season (April–September) retrieved from Landsat 8 imagery (2013–2018).

Figure 8 shows the average LST values through May–September, namely the months with more than 9 images each along the period 2013–2018. Figure 9, Figure 10 and Figure 11 illustrate the seasonal and, respectively, annual and multi-annual LST averages integrating all the images available over the period analyzed in this study. One can remark that the LST seasonality is perfectly captured by the Landsat data (Figure 8 and Figure 9). For example, June, July, and August (JJA) are the hottest months; the LST values decrease in spring (March, April, and May—MAM) and autumn (September, October, and November—SON); and winter is the coldest season (December, January, and February—DJF). Moreover, this is consistent with the thermal climate over the area of interest, as described in Section 2.1. Mean daily LSTs above 30 °C prevail from May to August, while 40–44 °C are common LSTs over the urban area of Bucharest during the daytime in July (Figure 8). In the central part of the city, the multiannual average LST values of the warm seasons range between 22 and 27 °C, and over the urban periphery the LST is 20–22 °C (Figure 11). The estimated SUHI intensity of 2.0–5.0 °C is in perfect agreement with previous work based on MODIS LST products [37].

One can remark that the spatial pattern of the LST (Figure 8, Figure 9, Figure 10 and Figure 11) is strongly connected with the land cover-land use characteristics of the urban area (Figure 1) for all the temporal scales tackled in this study. The warmest areas are concentrated in the central part Bucharest, overlapping the urban fabric districts with high building density, but spots with high LST values occur towards the edges of the administrative perimeter too, over industrial or residential urban fabric. The water and forest surfaces may be 8–10 °C colder than the urban fabric in terms of the multi-annual average (Figure 11).

**Figure 12:**
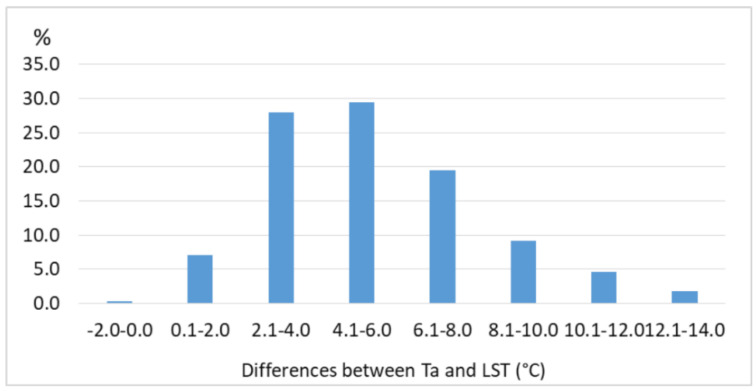
Frequency of the differences between Ta and the corresponding Landsat 8 LST values retrieved over the pixels containing the Bucharest weather stations (2013–2018).

**Figure 13:**
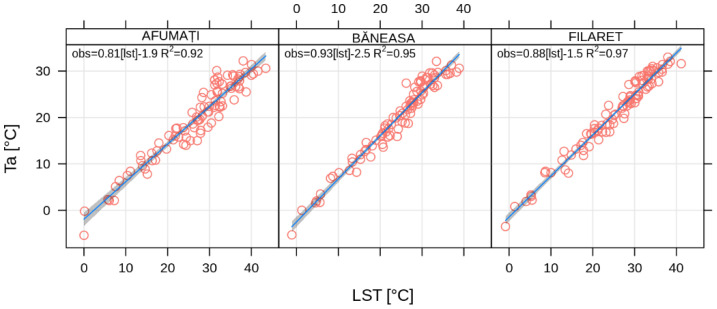
Ta versus LST values retrieved from Landsat 8 over the pixels corresponding to the Bucharest weather stations (2013–2018).

In order to confirm the accuracy of the method and the reliability of the results, the Landsat 8 LST values were compared with the corresponding air temperature retrieved at the same time of the day from the pixels containing the three WMO meteorological stations of Bucharest (i.e., București-Afumați, -Băneasa, and -Filaret). According to Jin and Dickinson (2010) [38], the difference LST–Ta is higher at noon (i.e., 15 °C), and lower by nighttime (i.e., <5 °C), while Mbuh et al. (2019) [39] found differences of up to 5 °C in Chicago and Minneapolis, based on Landsat 4, 5, and 7 images from 1984–2016. For Bucharest, the LST values retrieved from Landsat 8 OLI and TIRS over the pixels corresponding to the weather stations București-Filaret, -Băneasa, and -Afumați are higher than Ta, with 0.1 to 4.0 °C in 30% of the cases, and 4.1 to 6.0 °C in almost 30% of the cases (Figure 12). One can notice a strong correlation between LST and Ta, with Pearson’s correlation coefficients (R^2^) above 0.9 over each of the three meteorological stations analyzed here (Figure 13).

**Figure 14:**
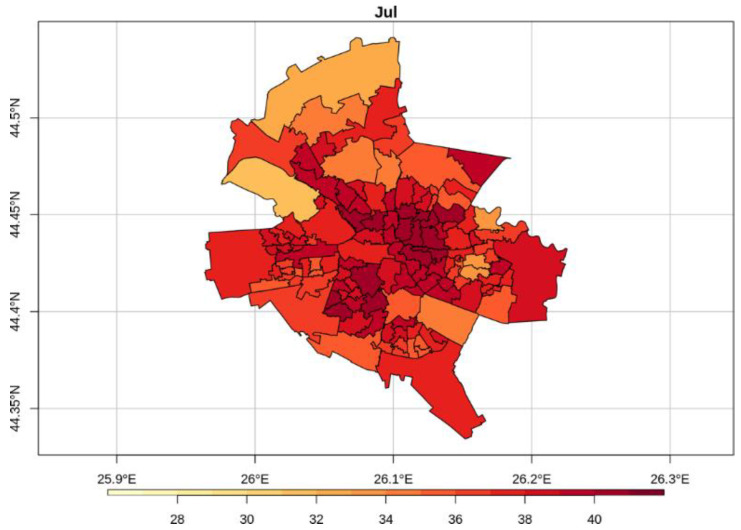
Daytime average Landsat 8-based LST (°C) over census units of Bucharest in July (2013–2018).

The gap-filled high-spatial-resolution LST data set based on the Landsat 8 imagery can be combined with other information and derive products that are very useful in different applications. For example, the distribution of the LST over census units of Bucharest (Figure 14) is fundamental information for assessing the thermal hazard risk at a fine scale, and it will be investigated as a follow-up of this study. Such details cannot be retrieved unless high-spatial-resolution data, such as the one obtained from gap-filling Landsat 8 imagery, is available.

One could summarize several clear benefits and inherent limitations related to the DINEOF-based gap filling of satellite imagery data, i.e., Landsat 8. The main benefits consist of (1) the delivery of a data set at high spatial resolution, (2) with full coverage over remote and heterogeneous areas where in situ weather stations’ measurements are lacking or are not continuous, which are (3) obtained at a relatively low cost for the end user [40]. The main advantage of using the DINEOF gap-filling procedure is that the method is easily applicable and does require supplementary variables (4). The coarse temporal resolution when using Landsat 8 imagery is an insurmountable barrier for some applications, such as operational forecasting, monitoring the rapid development of phenomena, or analysis of nighttime processes. However, the Landsat 8 data sets can be used for climate monitoring or risk studies related to more stable variables, such as LST [41].

## 4. Conclusions

Gap-free satellite remote sensing products at a fine resolution may be an excellent compromise between the need for full spatial coverage and temporal continuity of climate data in urban areas. The results of this study demonstrated that 30-m-spatial-resolution Landsat 8 imagery can be extremely useful for retrieving the LST in urban areas, despite the spatial and temporal discontinuity of the data sets. Although missing data, due to factors like cloudiness or a limited number of satellite passages, has been an important barrier for the proper use of Landsat LST in urban climate research, this study employed an efficient solution for obtaining more extended and better data sets, regarding both temporal and spatial coverage. This is an important advantage, especially in remote or heterogeneous areas, where filling the observational spatiotemporal gaps in data becomes more crucial [40]. This study demonstrated the advantage of applying the DINEOF method for filling LST gaps in satellite imagery in order to take full advantage of the high spatial resolution of the Landsat 8 data sets.

The DINEOF gap filling algorithm was applied to each Landsat 8 image with at least 40% valid LST values over Bucharest (Romania), and generated a consistent data set of 94 images with complete spatial coverage in the urban area through 2013–2018. The DINEOF procedure was validated by filling artificially created gaps in Landsat 8 images, and evaluating the results against the initial values in terms of statistic parameters, such as the correlation, RMS error, and variance. The returned LST values corresponding to the artificial gaps were found to be very well correlated and statistically consistent with the original data, pledging for an efficient gap-filling procedure. The land cover biases the performance of the gap filling, and the method performed slightly better for natural land cover categories, very likely due to their higher temperature homogeneity, but the results were very similar for all the categories.

The climatic analysis of the gap-free LST data set illustrated the role of the geographical setting and urban land-cover on the local climate. The comparison between LST and Ta at three WMO stations monitoring the climate of Bucharest (i.e., București-Afumați, -Băneasa, and -Filaret) returned strong correlation coefficients (R^2^ > 0.9) and approximately 70% of the differences were less than 6 °C, in very good agreement with the previous studies.

Further research may be envisaged aiming to complete this study with other data retrieved from sources like the updated Landsat 8, previous Landsat or other missions, ground-based data, modelling outputs, and ancillary data. Relevant improvements of the outputs in terms of the accuracy may be pursued by more complex validation campaigns using ground-based LST measurements and other satellite products. The LST distribution over census units of Bucharest exemplifies that by combining (1) high-spatial-resolution Landsat 8 images, with full urban coverage, and (2) detailed ground information, one can derive very useful products and applications.

## Figures and Tables

**Table 1:** Characteristics of several satellite remote sensing products delivering LST data.

Sensor	Satellite	Spatial Resolution	Temporal Resolution	Time Span
SEVIRI	MSG	3 to 5 km	15-min	1983 to date
AVHRR	NOAA	1.1 km	2 images/24 h	1981 to date
MODIS	Terra/Aqua	1 km	4 images/24 h	2000/2002 to date
SLSTR	Copernicus Sentinel-3	1 km	1 image/24 h	2017 to date
TM, ETM+, OLI, TIRS	Landsat 4, 5, 7, 8	30 m (resampled)	1 image/8 or 16 days	1982 to date

**Table 2:** Number of images used and average percentage of data coverage over the study area.

Year	2013	2014	2015	2016	2017	2018	Total
Number of images	13	14	13	21	19	14	94
Average percentage of data coverage (%)	91.4	94.7	93.0	88.5	90.0	90.7	91.4

**Table 3:** Percentage of LST data within the study area available from the selected Landsat 8 images.

**Percentage of Data Coverage (%)**	40.1–50.0	50.1–60.0	60.1–70.0	70.1–80.0	80.1–90.0	90.1–100.0
**Number of images**	2	0	1	7	16	68

**Table 4:** Summary statistics of the raw and DINEOF gap-filled LST data sets.

Statistical Parameter	Raw Data Set LST (°C)	Gap-Filled Data Set LST (°C)	Difference between Raw and Gap-Filled Data Sets (°C)
Minimum	−0.9	−2.6	1.7
1st Quartile	21.5	21.9	−0.4
Median	26.8	26.9	−0.2
Average	26.2	26.5	−0.3
3rd Quartile	32.7	33.0	−0.3
Maximum	53.6	51.3	2.3
